# Personalised Warfarin Dosing in Children Post-cardiac Surgery

**DOI:** 10.1007/s00246-019-02215-y

**Published:** 2019-10-05

**Authors:** Basma Zuheir Al-Metwali, Peter Rivers, Larry Goodyer, Linda O’Hare, Sanfui Young, Hussain Mulla

**Affiliations:** 1 School of Pharmacy, De Montfort University, Leicester, UK; 2 Department of Pharmacy, University Hospitals of Leicester, Glenfield Hospital, Groby Road, Leicester, LE3 9QP UK; 3 College of Pharmacy, University of Baghdad, Baghdad, Iraq; 4 East Midlands Congenital Heart Centre, University Hospitals of Leicester, Leicester, UK; 5 Department of Infection, Inflammation and Immunity, University of Leicester, Leicester, UK

**Keywords:** Warfarin, Personalised dosing, Pharmacokinetics, Pharmacodynamics

## Abstract

Warfarin dosing is challenging due to a multitude of factors affecting its pharmacokinetics (PK) and pharmacodynamics (PD). A novel personalised dosing algorithm predicated on a warfarin PK/PD model and incorporating CYP2C9 and VKORC1 genotype information has been developed for children. The present prospective, observational study aimed to compare the model with conventional weight-based dosing. The study involved two groups of children post-cardiac surgery: Group 1 were warfarin naïve, in whom loading and maintenance doses were estimated using the model over a 6-month duration and compared to historical case-matched controls. Group 2 were already established on maintenance therapy and randomised into a crossover study comparing the model with conventional maintenance dosing, over a 12-month period. Five patients enrolled in Group 1. Compared to the control group, the median time to achieve the first therapeutic INR was longer (5 vs. 2 days), to stable anticoagulation was shorter (29.0 vs. 96.5 days), to over-anticoagulation was longer (15.0 vs. 4.0 days). In addition, median percentage of INRs within the target range (%ITR) and percentage of time in therapeutic range (%TTR) was higher; 70% versus 47.4% and 83.4% versus 62.3%, respectively. Group 2 included 26 patients. No significant differences in INR control were found between model and conventional dosing phases; mean %ITR was 68.82% versus 67.9% (*p* = 0.84) and mean %TTR was 85.47% versus 80.2% (*p* = 0.09), respectively. The results suggest model-based dosing can improve anticoagulation control, particularly when initiating and stabilising warfarin dosing. Larger studies are needed to confirm these findings.

## Introduction

Warfarin, the most widely prescribed oral anticoagulant, represents a major challenge to successful therapy in children. The drug is indicated for the prevention of thromboembolism associated with underlying disorders such as congenital heart disease with or without mechanical prosthetic valves, cancer, renal disorders and long-term total parenteral nutrition [1, 2]. However, maintaining the target therapeutic INR is intensely challenging because of a multitude of factors, genetic and non-genetic, affecting warfarin pharmacokinetics (PK) and pharmacodynamics (PD) and hence dose requirements and response [3–6]. The results from a large cohort study of 319 children treated with warfarin showed that the proportion of International Normalised Ratio (INR) measurements within the target range 2.0–3.0 and 2.5–3.5 was only 47% and 61%, respectively [7]. The overall incidence of major bleeding events was shown to be 0.5% per patient year [7], with patients with mechanical heart valves having a higher incidence of up to 4% per patient year [8].

To individualise warfarin dosing in children and hence improve treatment outcomes, a warfarin dose individualisation kinetic/pharmacodynamic (K/PD) model was developed in children and included age, CYP2C9 and VKORC1 genotypes as covariates [9]. The model is an extension of a previous K/PD model in adults that describes the relationship between warfarin dose and INR response to overcome the lack of plasma warfarin concentration (PK) data [10]. The predictive performance of the bridged paediatric model using a Bayesian approach was evaluated in a cohort of 49 children treated with warfarin. The model was able to predict ideal maintenance doses (within ± 20% of the observed doses) in 41% of patients, which increased to 70% when 3 or more INR observations were available [9].

To permit user-friendly warfarin dose individualisation in children, the Hamberg K/PD model combined with a Bayesian forecasting algorithm has been integrated in a Java-based decision support tool [11]. The tool can be used for a priori prediction of initial doses by entering information on bodyweight, baseline and target INR, and CYP2C9/VKORC1 genotypes if available, and subsequently, by adding information about previous doses and INR observations, the tool estimates (revises) a posteriori maintenance doses.

To date, only one prospective clinical study of children has been conducted to compare genotype-guided warfarin dose individualisation with the conventional, weight-adjusted dosing approach [12]. Genotype-guided dosing was found to significantly decrease the time to stable dose and hospital stay days [12]. Warfarin dose individualisation in children using PK/PD models has not been investigated. The aim of the current study was to compare, prospectively, warfarin dose individualisation using the Hamberg K/PD model-based decision support tool, with the conventional, weight adjusted approach. The study also aimed to explore the views of children/parents and healthcare providers about the usual warfarin dosing/monitoring process as well as their views about model-based approach to warfarin dosing. The findings from this qualitative aspect of this study are not included here.

## Materials and Methods

The study site was Glenfield Hospital in Leicester, England and the study was approved by the East Midlands Nottingham Ethics Committee (15/EM/0325). Written, informed consent was obtained from parents/legal guardians and assent from children over 12 years of age.

### Validation of the Hamberg K/PD Model

To validate the Hamberg K/PD model (‘model’), retrospective cohort data were extracted from a database of children aged from birth to 18 years archived at Glenfield Hospital. The data collected included date of birth, gender, ethnicity, weight, indication, target INR range, date started, doses and the corresponding INR observations.

The model was used as a hypothetical tool to predict each patient’s warfarin maintenance doses and these were then compared with the actual doses prescribed by clinicians. The assessment was conducted during the period children were observed to have stable maintenance warfarin dosing, defined as at least three consecutive INR measurements in the target therapeutic INR range over a period of at least four weeks with no change in warfarin dose [9].

### Prospective Study

Prospective evaluation of the model was undertaken in two groups of post-operative cardiac children. Group 1 included patients starting warfarin treatment for the first time post-cardiac surgery (*warfarin naïve)*. These include children presenting for Fontan procedure or replacement of the mitral or aortic valves and were identified pre-operatively and on one occasion on the ICU post-operatively, where the decision to replace the heart valve was made intra-operatively. Initial and maintenance warfarin doses were estimated using the model over a 6-month duration and compared with historical case-matched controls dosed according to the conventional approach. Cases were matched according to age (± 1.0 year), indication and target INR range. Concomitant medications used in the post-operative period were comparable between the case and control groups.

Group 2 consisted of post-cardiac surgical children who were already established on long-term (maintenance) warfarin treatment and were identified from the hospital database based on age, indication for warfarin therapy and target INR range. Group 2 patients entered a randomised crossover study comparing model-estimated dose adjustments (Model phase) with the conventional approach (Doctor phase). No washout period between the two phases was included in this study as patients required continuous oral anticoagulation. Therefore, patients continued on the last dose prescribed in the preceding phase until the designated day for INR measurement (a maximum of 3 weeks). After the INR measurement was reported, determination of dose and date of next INR measurement was made using the second (new) phase of treatment. Warfarin treatment stopped a few days prior to undergoing procedures like cardiac catheterisation or dental procedures and resumed immediately afterwards. The follow-up period was 6 months in each phase of treatment, i.e. a total period of 12 months. Study outcomes in the two phases of treatment were compared.

The primary outcomes of the study were the time taken to achieve first therapeutic INR (Group 1 only), time taken to achieve stable anticoagulation (Group 1 only) and the percentage of INR measurements (%ITR) and percentage of time in target therapeutic range (%TTR).

Stable anticoagulation was defined as at least three consecutive INR measurements in the target therapeutic range (TTR) over a minimum period of four weeks with no change in warfarin dose [9].

The percentage of time in therapeutic range (%TTR) was determined by linear interpolation [13].

#### Genotyping

Mouth swabs for genotyping CYP2C9*2, CYP2C9*3 and VKORC1 − 1639G>A alleles was undertaken in Group 1 patients either pre-operatively or post-operatively prior to the initiation of warfarin treatment. For Group 2 patients, mouth swabs were obtained on the day of their hospital visits. Genotyping results were not always available upfront in Group 2 patients but in any case, were not necessary, as the model is capable of predicting the phenotype based on the previous warfarin doses and INR values of the patient. Nevertheless, all genotyping results were used in final data analysis. Genotyping was performed using a point of care genotype testing instrument, ParaDNA® (LGC).

#### INR-Monitoring and Calculation of Warfarin Doses

In the majority of patients (in both groups), warfarin therapy was monitored using home INR-monitoring machines; only a few families attended hospital for INR monitoring. Families who used the home-monitoring machines telephoned the INR test result together with information about any intercurrent illness and/or medication use that may affect anticoagulation stability.

In Group 1, initial warfarin doses were estimated using the model. Excel files were created for each individual patient and after every INR observation (feedback), the patient’s model parameters were re-estimated with subsequent prediction of the tailored maintenance dose.

When Group 2 patients entered the Model phase, Excel files were created using the last two-month history of warfarin dosing and INR monitoring (at least 3 INR observations were used to transition into the model phase) and individual model parameters estimated with subsequent maintenance dose prediction. The files were updated after every INR observation, model parameters re-estimated and maintenance doses subsequently revised where necessary.

Model-estimated doses were rounded to practical doses which were then reviewed and prescribed by the doctors.

Warfarin doses in the Group 1 control patients and during the Doctor phase for Group 2 patients were prescribed by a paediatric cardiology team member, according to the unit protocol; loading dose of 0.2 mg/kg and maintenance dose adjusted according to response.

#### Sample Size

No sample size calculation was made for Group1; the final number of patients recruited was limited by study feasibility over a 14-month period.

Sample size for Group 2 was estimated using the method for paired continuous data [14]. The primary outcome measure, %ITR was utilised and in the existing database of children at the hospital was determined to be mean (SD) 54.1% (16.9). Based on a clinically relevant effect size (difference between model-based and conventional method) of 11% (to increase the proportion of within-range INR measurements to 65%), a standardised effect size (computed using the SD estimate from the existing database) was derived. Hence for 80% power and two-sided 5% significance level, a sample size of 25 was estimated. To allow for some patients dropping out, a total of 30 patients were to be recruited.

#### Data Analysis

Model accuracy was evaluated by calculating the difference between model-predicted and observed doses, and the results were expressed as prediction error (PE):

$${\text{PE}} = \frac{{\left( {{\text{predicted dose}} - {\text{observed dose}}} \right)}}{{\text{observed dose}}}$$

The bias (mean PE) and precision (root mean squared error) were also calculated. Clinical accuracy was evaluated by calculating the percentage of patients in which the model-predicted dose was ideal (within 20% of the observed dose), under-predicted (at least 20% below the observed dose) or over-predicted (at least 20% above the observed dose) [9].

For Group 1, characteristics of the study population and study outcomes were summarised using descriptive statistics. For Group 2, primary endpoints were compared using paired sample t test or Wilcoxon test as appropriate. The effect of genetic (CYP2C9, VKORC1 genotype) and non-genetic (age groups, gender, ethnicity, indication of warfarin, target INR range) variables on primary endpoints was also evaluated using an independent sample t test, Mann–Whitney test, analysis of variance (ANOVA) or Kruskal–Wallis test as appropriate. A p value of less than 0.05 was considered to be significant.

## Results

### Retrospective Study to Validate the Hamberg K/PD Model

Sixty patients with complete treatment and monitoring histories were used to evaluate the model. Demography and characteristics of the model validation cohort are presented in Table 1.

**Table 1:** Characteristics of the study population

Indicator	Model validation cohort *N* = 60	Prospective study cohorts		
		Group 1		Group 2 *N* = 26
		Case subjects *N* = 5	Control subjects *N* = 5	
Age^a^ (years), median (range)	16.75 (8.4–66.6)	6 (3.8–8.9)	5.3 (3.4–9.3)	9.0 ± 4.8 (1–17.3)^b^
Weight (kg), median (range)	5.2 (1–15.9)	16 (15.4–30.3)	17 (16–36.5)	24.9 (9.5–62.8)
Gender [*N* (%)]				
Male	39 (65)	–	3 (60)	18 (69.2)
Female	21 (35)	5 (100)	2 (40)	8 (30.8)
Ethnicity [*N* (%)]				
White	43 (71.7)	2 (40)	5 (100)	20 (76.9)
Asian	8 (13.3)	3 (60)	–	4 (15.4)
Other^c^	8 (13.3)	–	–	2 (7.7)
Missed	1 (1.7)	–	–	–
CYP2C9 genotype [*N* (%)]				
*1/*1	NA	5 (100)	NA	16 (61.5)
*1/*2		–		6 (23.1)
*1/*3		–		3 (11.5)
Missing		–		1 (3.8)
VKORC1 genotype [*N* (%)]				
G/G	NA	3 (60)	NA	12 (46.2)
G/A		2 (40)		14 (53.8)
Indication for warfarin [*N* (%)]				
Fontan	41 (68.3)	3 (60)	3 (60)	20 (76.9)
MVR	6 (10)	2 (40)	2 (40)	5 (19.2)
AVR	10 (16.7)	–	–	1 (3.8)
Other^d^	3 (5)	–	–	–
Target INR range [*N* (%)]				
2.0–3.0	23 (38.3)	3 (60)	3 (60)	12 (46.2)
1.5–2.5	16 (26.7)	–	–	7 (26.9)
2.5–3.5	8 (13.3)	2 (40)	2 (40)	4 (15.4)
2.0–2.5	7 (11.7)	–	–	1 (3.8)
Other^e^	6 (10)	–	–	2 (7.7)

*NA* not available, *MVR* mitral valve replacement, *AVR* aortic valve replacement

^a^Age at enrolment

^b^Mean ± SD (range)

^c^Other ethnicity: mixed White and Black, mixed White and Asian, Black, mixed White and Black Caribbean and Middle Eastern

^d^Other indications: Kawasaki disease and stroke

^e^Other target INR ranges 1.8–3.0, 1.5–3.0, 1.5–3.5, 2.0–3.5, 2.5–3.0, 3.0–3.5 and 3.0–4.0

Seventy percent of the dose predictions were ideal, i.e. within ± 20% of the observed doses; 25% of the predicted doses were underestimated and 5% were overestimated. The bias was − 0.10 which implies an overall dose under-prediction of 0.1 mg. The precision was 0.19, implying an imprecision in dose predictions of 19%.

### Prospective Study

#### Group 1: Warfarin-Naive Patients

Patient recruitment occurred between October 2015 and December 2016. Nine consecutive patients were screened, and five consented to participate. The characteristics of the Group 1 case and historical control patients are summarised in Table 1. Results of the study outcomes for Group 1 case and controls are shown in Table 2.

**Table 2:** Results of the study outcomes for Group 1 case and control subjects

Outcome	Control (*n* = 5)	Case (*n* = 5)
Time to first therapeutic INR (days)	2 (1–3)	5 (2–6)
Time to stable anticoagulation (days)	96.5 (24–138)^a^	29 (9–87)^b^
Time to over-anticoagulation (INR ≥ 4.0) (days)	4 (1–14)	15 (4–17)^b^
%ITR	47.4 (43.6–55.1)	70 (53.2–76.9)
%TTR	62.3 (38.2–71.3)	83.4 (69–84.4)
Number of dose changes	21 (12–36)	20 (8–50)
Frequency of INR measurements (per month)	6.3 (4–11.5)	5 (3.8–13.2)
No. of INR values ≥ 4.0	2 (2–6)	2 (0–11)^b^
No. of INR values ≥ 5.0	2 (1–3)	0 (0–2)^c^

Values are expressed as median (range)

^a^*n* = 4

^b^*n* = 3

^c^*n* = 2

A total of 436 INR measurements were collected from case and control subjects. Four (1.9%) out of 212 dose recommendations made by the model were overridden by doctors.

**Fig. 1:**
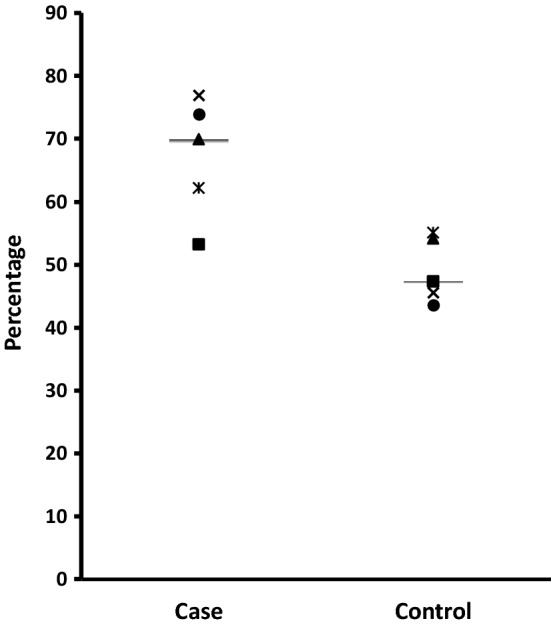
The percentage of measured INR in the target therapeutic range (%ITR) in Group 1 subjects (bar represents the median subject)

**Fig. 2:**
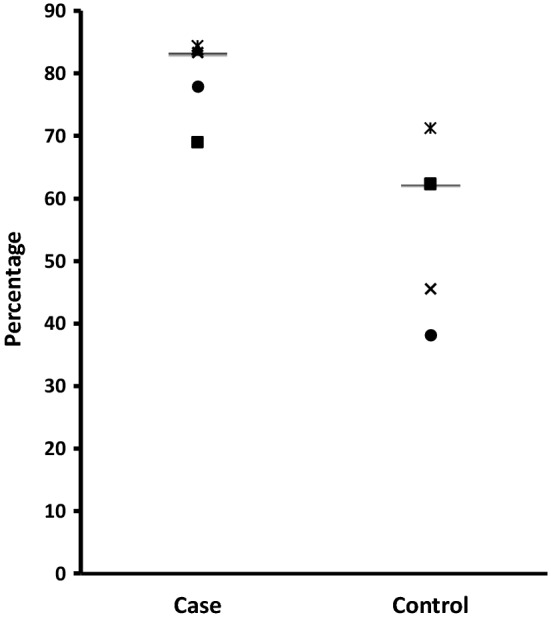
The percentage of time in the target therapeutic range (%TTR) in Group 1 subjects (bar represents the median subject)

The median time to achieve the first INR values within the target therapeutic range was 5 days for the case subjects compared to 2 days for control subjects. Two case patients and one control patient did not achieve stable anticoagulation during the 6-month period of follow-up. The median time to stability for the remaining three case patients was 29 days, compared to 96.5 days for the four control patients. Two of the case patients (aged 5.4 and 6 years) who did not achieve stability were anticoagulated for mechanical mitral valves, whereas the control patient (aged 9.3 years) who did not achieve stability was anticoagulated for Fontan circulation. The percentage of INRs and time in therapeutic range was higher in the model dosed patients: median %ITR and %TTR for the case and control subjects was 70% versus 47.4% and 83.4% versus 62.3%, respectively (Figs. 1, 2). There was a prolongation of time to over anticoagulation and reduced likelihood of over anticoagulation in the model dosed patients: for the case and control subjects, median time to first INR value ≥ 4.0 was 15 days versus 4 days, respectively and median number of INR values ≥ 4.0 and ≥ 5.0 was 2 versus 2 and 0 versus 2, respectively. Warfarin treatment was withheld in four control subjects on five occasions, including one occasion when vitamin K was administered, compared to none in the case group.

IV heparin was used in two case subjects on one occasion compared to two occasions of IV heparin use in one control subject and one occasion of LMWH use in one control subject.

The median frequency of INR measurements and dose alterations for case versus control subjects was 5 versus 6.3 and 20 versus 21 measurements/month, respectively.

#### Group 2: Warfarin Established Patients

Forty-eight patients were screened, and 29 patients were enrolled; 26 patients completed the follow-up period and were included in the analysis. One patient died because of deterioration of his medical condition, one parent withdrew consent and one patient was withdrawn following a bioprosthetic replacement of his mechanical valve. The characteristics of Group 2 patients are summarised in Table 1.

A total of 1073 INR measurements were collected during both phases of treatment over a total follow-up period of 26 patient years. Twenty-two (3.9%) out of 571 model recommended doses were overridden by doctors.

There was no significant difference in target INR attainment between the Model and Doctor phases: mean (SD) %ITR 68.8 (19.8) versus 67.9 (23.2) % (*p* = 0.84) and %TTR 85.5 (13.0) versus 80.2 (18.0)% (*p* = 0.09), respectively. Warfarin therapy was significantly disrupted in 5 patients (4 during Model phase and 1 during Doctor phase). This was due to surgical procedures (cardiac catheterization or dental) and/or periods of illness, where warfarin treatment was stopped for a period of time and then resumed. After excluding these cases, the %TTR during the Model phase was significantly higher than the Doctor phase; 87.0 (12.8) versus 78.5 (17.2) %, respectively, *p* = 0.03. Although the %TTR was higher during the Model phase irrespective of indication, it was significant in Fontan patients (90.4% vs. 80.9%, *p* = 0.02) but not in mechanical valve patients (72.6% vs. 68.5%, *p* = 0.51).

The median frequency of INR measurements per month was slightly higher during the model phase; 2.3 versus 1.9 measurements/month, respectively (*p* = 0.08). The median frequency of dose alterations was significantly higher during the model phase (6.5 vs. 2.5, *p* = 0.02).

There was no significant difference between the two phases of treatment in the number of INR values ≥ 4.0 (*p* = 0.9) and those ≥ 5.0 (*p* = 0.8), including when stratified by indication (Fontan or mechanical valve). Warfarin treatment was withheld in 2 patients during the Model phase and 3 patients during the Doctor phase (including the administration of vitamin K on one occasion). Similarly, to address low INRs, IV heparin was used in 4 patients on six occasions during the Model phase compared with 3 patients on four occasions during the Doctor phase. LMWH was used in one patient on one occasion during the Model phase. However, this was a teenager with poor treatment adherence and concomitant alcohol intake which led to a decrease in INR.

#### Influence of Patient-Related Factors on Dose and Target INR Attainment in Group 2 Patients

There were no differences in target INR attainment associated with age, gender or ethnicity (Table 3). Patients with mechanical valves had significantly lower success of achieving target therapeutic range than those with Fontan circulation (72.3 vs. 55.3%ITR, *p* = 0.04; 88.5 vs. 65.6%TTR, *p* = 0.04).

**Table 3:** Descriptive statistics and *p* values of the effect of genetic and non-genetic variables on daily warfarin dose, %ITR and %TTR in Group 2 patients

	*N*	mg/kg/day Median (range)	*p* value	%ITR Mean (range)	*p* value	%TTR Median (range)	*p* value
Age groups (years)							
1–5	10	0.2 (0.1–0.4)	0.17^a^	58.75 (36.4–78)	0.1^b^	83.2 (52.9–90.5)	0.19^a^
6–10	6	0.1 (0.1–0.2)		72.63 (57.7–100)		84.75 (75.7–100)	
11–18	10	0.1 (0.1–0.2)		75.41 (43.8–100)		90.98 (69.1–100)	
Gender							
Male	18	0.2 (0.1–0.4)	0.11^d^	67.13 (36.4–100)	0.62^c^	86.15 (52.9–100)	0.94^d^
Female	8	0.1 (0.1–0.2)		71.13 (43.8–100)		86.1 (69.1–100)	
Ethnicity							
White	20	0.1 (0.1–0.4)	0.35^a^	67.97 (36.4–100)	0.98^b^	86.15 (52.9–100)	0.8^a^
Asian	4	0.2 (0.1–0.2)		68.93 (57.7–77.7)		87.4 (77.8–95.6)	
Other	2	0.15 (0.1–0.2)		71.05 (50–92.1)		88.3 (82.2–94.5)	
Indication							
Fontan	20	0.1 (0.1–0.2)	0.16^d^	72.30 (36.4–100)	0.04^c^	88.45 (59.3–100)	0.04^d^
Mechanical valves	6	0.2 (0.1–0.4)		55.23 (44.1–82.3)		65.55 (52.9–94.0)	
Target INR range							
1.5–2.5	7	0.1	0.07^a^	79.74 (63.4–100)	0.17^b^	88.95 (75.7–100)	0.21^a^
2.0–3.0	12	0.1 (0.1–0.2)		67.45 (36.4–94.5)		85.75 (59.3–99.8)	
2.5–3.5	4	0.2 (0.1–0.4)		55.25 (44.1–82.3)		63.28 (52.9–94.0)	
Other	3	0.2 (0.1–0.2)		62.9 (45.4–85.7)		77.75 (61.5–97.1)	
CYP2C9 genotype							
*1/*1	16	0.1 (0.1–0.2)	0.56^d^	72.15 (36.4–100)	0.28^c^	88.45 (57.0–100)	0.36^d^
*1/x	9	0.1 (0.1–0.4)		63.66 (43.8–100)		80.5 (52.9–100)	
VKORC1 genotype							
G/G	12	0.2 (0.1–0.4)	0.01^d^	61.50 (44.1–85.7)	0.08^c^	83.25 (52.9–97.1)	0.11^d^
G/A	14	0.1 (0.1–0.2)		74.23 (36.4–100)		89.4 (59.3–100)	

^a^Kruskal-Wallis test

^b^ANOVA test

^c^Independent sample t test

^d^Mann–Whitney test

Patients with VKORC1 wild type allele G/G required statistically significantly higher doses than those with G/A genotype (0.2 vs. 0.1 mg/kg/day, *p* = 0.01) whilst dose was similar for patients with CYP2C9 wild type (*1/*1) and variant alleles (*1/*2 and *1/*3) (*p* = 0.56). There was no difference in %ITR or %TTR between CYP2C9 variant (*1/*2 and *1/*3) wild type alleles (63.7 vs. 72.2%ITR, *p* = 0.28; 80.5 vs. 88.5%TTR, *p* = 0.36). There was also no difference between variant VKORC1 (G/A) and wild type allele (74.2 vs. 61.5%ITR, *p* = 0.08; 89.4 vs. 83.3% vs. %TTR, *p* = 0.11).

## Discussion

Warfarin is the most widely used oral anticoagulant in children with congenital heart disease, but dosing is challenging because of considerable inter- and intra-individual variability in its PK/PD and the effect of genetic polymorphisms. Genotype-guided warfarin dosing has previously been investigated in children and shown to decrease time to stable dose and hospital stay days but found no difference in time to first therapeutic INR, time before over-anticoagulation occurred and bleeding events, compared with the standard dosing approach [12]. In adults, genotype-guided warfarin dosing has shown to increase the proportion of time in the therapeutic range, fewer incidents of over-anticoagulation and shorter time to therapeutic INR than the standard dosing approach [15].

Population PK/PD models account for factors contributing to the variability in exposure and response and combined with Bayesian forecasting algorithms have emerged as a potent tool for personalising drug therapy. Personalised dosing is a concept that recognises each individual has unique PK and PD characteristics, governing the time course of drug effect, and pivotal to optimising therapy. Knowledge of the individual’s PK/PD parameters is therefore key to individualising drug doses and improving treatment response. PK/PD model-based warfarin dosing has been investigated in adults and shown to result in significantly higher proportion of time in target therapeutic range, lower proportion of out-of-range INR values and shorter time to first therapeutic INR and stable anticoagulation when compared to the genotype-guided dosing [16]. In this report, we have presented for the first time, the results of dosing children with a warfarin K/PD model combined with Bayesian forecasting algorithm.

The predictive performance of the Hamberg K/PD model and hence suitability for implementation in the prospective study was first validated in a cohort of 60 post-operative cardiac children, of predominantly Caucasian and Asian descent, on long-term warfarin treatment in our institution. The model, previously tested in Swedish children, was shown to perform well with minimal bias and reasonable precision and provided confidence for use in a routine clinical setting.

The prospective study evaluated two aspects of model-based warfarin dosing. Group 1 patients, in whom oral warfarin therapy was initiated for the first time, assessed whether the model with age, weight and genotype as inputs but no previous INR observations, could improve therapy in the initial phase, particularly attainment and control of INR in the target range. Group 2 assessed model performance in the maintenance phase*,* in patients already established on warfarin therapy and with multiple previous INR observations.

Five patients starting warfarin treatment for the first time after congenital heart surgery were recruited in Group 1. The results showed that compared to case-matched controls, model-based warfarin dosing resulted in a 48% increase in INR measurements within the target therapeutic range (%ITR) and a 34% increase in percentage of time within the therapeutic range (%TTR). In addition, model-based warfarin dosing resulted in fewer over-anticoagulated patients and when it did occur, a longer time to over-anticoagulation and a shorter time to reach stable anticoagulation. The time to reach a therapeutic INR was 3 days longer using the model-based dosing approach when compared to the conventional dosing approach. These differences largely reflect differences between the two dosing approaches. The usual clinical practice is to start with a loading dose of 0.1–0.2 mg/kg (maximum 10 mg) which may be repeated if the subsequent INR value is between 1.1 and 1.4. In contrast, the model predicts the initial warfarin dose based on typical population parameter estimates and the individual patient’s covariates (age, weight and CYP2C9 and VKORC1 genotypes). Subsequent dose adjustments are made after the INR feedback is obtained from the patient. These results are similar to a previously reported genotype-guided dosing study in children [12].

In patients already established on warfarin (Group 2), small non-statistically significant improvements in %ITR and the %TTR was observed during the model phase compared to the doctor phase, mean difference 0.92% (*p* = 0.84) and 5.27% (*p* = 0.09), respectively. However, after excluding 5 patients who experienced significantly disrupted warfarin therapy, the %TTR of the model-based approach was significantly higher than that of the traditional approach (*p* = 0.03).

The greater improvement in %TTR compared to %ITR may be attributed to the difference between the two approaches in calculating successful therapeutic range attainment. The %ITR is simply the proportion of INR values within the target range whereas the %TTR allocates an INR value for each day between subsequent INR tests according to the linear interpolation approach [13]. Although having the advantage of being easy to calculate, the %ITR underestimates the time in therapeutic range in children, particularly in the periods of instability during which the INR is tested more frequently for dose adjustment. Therefore, the %TTR can provide a better estimation of the time in therapeutic range in this population [17]. In addition, in this study there were many times where the INR measurements were very slightly above or below the target range and thus considered out-of-range. This led to an underestimation of the time in therapeutic range calculated as %ITR whereas the %TTR provided a better estimation.

Surprisingly, although the %ITR estimated in our retrospective cohort was 54.06% (similar to literature reports), during the prospective study a much higher %ITR of 67.9% was recorded during the Doctor phase. It is possible that as a consequence of the study, clinicians were more diligent and careful in their prescribing and monitoring of therapy and consequently non-significant differences emerged from the two phases of treatment. Nevertheless, results from Group 1 and Group 2 suggest that K/PD model-based warfarin dosing has the largest impact when initiating and stabilising warfarin dosing. Once patients are stabilised on a warfarin maintenance dose, the benefits of model-based warfarin dosing are less certain.

The subgroup analysis suggests that improvement in %TTR during the model phase was more evident for patients with Fontan circulation and in groups who are described as being more challenging by healthcare professionals [18, 19]; children below 5 years of age (mean difference in %TTR 4.8%, *p* = 0.29), adolescents (mean difference in %TTR 2.3%, *p* = 0.58) and children with mechanical heart valves (mean difference in %TTR 4.1%, *p* = 0.51).

The frequency of INR measurements per month was comparable between the two dosing approaches, but the model-based approach was associated with lower levels of over-anticoagulation when compared to the conventional approach, although this was not statistically significant. However, the number of dose changes was statistically significantly higher in the model-based approach when compared to the conventional dosing approach. This reflected the method of dose estimation, whereby the model adjusts the dose to the mid-value of the target INR range and hence recommended dose changes for only slight, clinically insignificant changes in the INR. In comparison, during the Doctor phase of dosing, such small changes tended to be ignored and hence longer testing schedules with less frequent testing schedules were employed. The experience of individual doctors may have also had a role to play.

No effect of CYP2C9 genotype on dose requirements was observed, which is consistent with a previous report [6, 20–22]. In contrast, patients with VKORC1 wild type allele required significantly higher doses. There have been previous reports that CYP2C9 and VKORC1 genotype can influence the success of attaining target INR range particularly in the first 6 months of therapy, however no differences were found in this study, presumably a consequence of the small numbers in the genotype subgroups and the absence of any patients with homozygous variant alleles that require the lowest warfarin dose requirements [4].

A major limitation of the present study was the small number of warfarin naïve patients recruited in Group 1, precluding statistically valid comparisons between model and conventional dosing. However, this study was designed to be a preliminary investigation to assess the effectiveness of model-based warfarin dosing in children, including feasibility of implementing in clinical practice. Further studies with larger sample size that includes a greater proportion of children with mechanical heart valves and variant alleles of CYP2C9 and VKORC1 are required to assess the benefits of model-based warfarin dosing but also to provide a better understanding of the effects of genetic and non-genetic factors on warfarin dose requirement and time in therapeutic range. A limitation of Group 2 data was the lack of a wash-out period when crossing over from one phase to another. A wash-out period was not feasible in this study as children required constant anticoagulation with warfarin to prevent thromboembolic events. This may have affected the %ITR and %TTR, but any effect was likely to be minimal as most patients had a repeat INR within a week of transition, upon which warfarin dose management switched to the alternative method.

Despite the limitations of the present study, the results appear to support previous findings that PK/PD model directed warfarin treatment can improve the time in therapeutic range and reduce the risk of under- and over-dosing. Improved anticoagulation control with model-based warfarin dosing, by minimising above and below range INR values, could prove to be clinically advantageous by reducing the risk of bleeding and thrombosis. Furthermore, this study has shown that model-based warfarin dosing can be implemented in routine care of children. This was reflected in the fact that less than 4% of the model-predicted doses were overridden by prescribing doctors, suggesting an overall acceptance of health care professionals to the model-based dosing approach as a basis for predicting the most optimum doses of warfarin.

In conclusion, the preliminary results obtained from this study show that model-based personalised warfarin dosing could improve anticoagulation control in children after heart surgery, particularly during the initiation and stabilisation of warfarin therapy. However, the approach needs to be explored further in a larger randomised controlled study to confirm these preliminary results.
